# Blood outgrowth endothelial cells (BOECs) as a novel tool for studying adhesion of *Plasmodium falciparum*-infected erythrocytes

**DOI:** 10.1371/journal.pone.0204177

**Published:** 2018-10-09

**Authors:** Gertrude Ecklu-Mensah, Rebecca W. Olsen, Anja Bengtsson, Michael F. Ofori, Lars Hviid, Anja T. R. Jensen, Yvonne Adams

**Affiliations:** 1 Department of Immunology, Noguchi Memorial Institute for Medical Research, University of Ghana, Legon, Ghana; 2 Centre for Medical Parasitology at Department of Immunology and Microbiology (ISIM), Faculty of Health and Medical Sciences, University of Copenhagen, Copenhagen, Denmark; 3 Department of Infectious Diseases, Copenhagen University Hospital (Rigshospitalet), Copenhagen, Denmark; Université Pierre et Marie Curie, FRANCE

## Abstract

The lack of suitable animal models for the study of cytoadhesion of *P*. *falciparum-*infected erythrocytes (IEs) has necessitated *in vitro* studies employing a range of cell lines of either human tumour origin (e.g., BeWo and C32 cells) or non-human origin (e.g., CHO cells). Of the human cells available, many were isolated from adults, or derived from a pool of donors (e.g., HBEC-5i). Here we demonstrate, for the first time, the successful isolation of blood outgrowth endothelial cells (BOECs) from frozen stabilates of peripheral blood mononuclear cells obtained from small-volume peripheral blood samples from paediatric malaria patients. BOECs are a sub-population of human endothelial cells, found within the peripheral blood. We demonstrate that these cells express receptors such as Intercellular Adhesion Molecule 1 (ICAM-1/CD54), Endothelial Protein C Receptor (EPCR/CD201), platelet/endothelial cell adhesion molecule 1 (PECAM-1/CD31), Thrombomodulin (CD141), and support adhesion of *P*. *falciparum* IEs.

## Introduction

The vascular system is one of the largest organs in the human body and is the primary point of interaction between infected erythrocytes (IEs) and the infected host. The IEs utilize various endothelial receptors such as ICAM-1 (CD54), EPCR (CD201) and platelet glycoprotein 4 (CD36) to sequester and evade phagocytic destruction in the spleen [1–4]. The receptor interactions are mediated by the constituent Duffy binding-like (DBL) and cysteine rich-inter-domain (CIDR) domains of the parasite protein family *Plasmodium falciparum* erythrocyte membrane protein-1 (PfEMP1), encoded by the multi-gene *var* family (reviewed in[5]). Despite their diversity, the PfEMP1 proteins can be classified into groups A, B and C according to the 5’ un-translated region of the *var* genes, their genomic location, and their direction of transcription[6,7]. These groups have been associated with clinical presentation of malaria patients, with group A more commonly found among those with severe disease [2,8–12]. Biopsies and post-mortem samples from children diagnosed with cerebral malaria show multiple organs saturated with sequestered IEs, and disease severity is associated with increased parasite biomass; findings supporting the importance of cytoadhesion in the pathology of *P*. *falciparum* infections [11,12]. Current cell lines available for the study of the adhesive interactions between IEs and host endothelium are either non-human (CHO), cancer-derived (C32, BeWo etc.), or are commercially available human primary and/or immortalised cell lines (HBEC-5i) collected from multiple donors who do not correspond either in age or geography to the individuals most at risk of malaria (i.e. children <5 years old) [13–16].

Within the blood stream are a number of mononuclear circulatory cells, of which BOECs are a rare sub-population (1–4 cells/mL) [17–19]. BOECs are distinct from endothelial progenitor cells by their lack of prominin-1 (CD133; a marker for stem cells) expression [20], while they do express cell surface glycoprotein muc18 (CD146; a marker for endothelial cells) [21]. BOECs have previously been used in the studies of von Willebrand Factor disease [21] and neovascularization[22], but to our knowledge not in any malaria studies. Here we report the successful isolation and expansion of BOECs from small-volume samples obtained from paediatric malaria patients in a remote district hospital (Hohoe, Ghana) and cryopreserved in the field. The recovered BOECs supported the adhesion of IEs *in vitro*. We propose that these cells, which represent the microvasculature of naturally infected malaria patients, offer a minimally invasive and convenient means to study inter-individual differences in the endothelium of clinically relevant populations.

## Materials and methods

### Patient enrolment

*P*. *falciparum*-malaria patients were recruited during a two-week period in 2016 from the Hohoe Municipal Hospital in the Volta Region of Ghana. After informed consent had been obtained from a parent or a guardian, 34 children (<12 years-of-age) were enrolled and subjected to clinical investigation, collection of venous blood samples, and treatment according to national guidelines. Malaria symptoms were classified according to the World Health Organization criteria [23]. The study was approved by the Ethical Review Committee of the Ghana Health Services (file GHS-ERC 08/05/14).

### Blood outgrowth endothelial cell collection, cryopreservation, and culture

BOECs were isolated as previously described with some modifications [24]. Peripheral blood samples were collected from healthy adult Europeans (30 mL; n = 5) and Ghanaian children (2–8 mL; n = 34) in lithium heparin tubes (Becton Dickson). Blood samples were diluted 1:1 with PBS, and peripheral blood mononuclear cells (PBMCs) isolated by density-gradient centrifugation over Histopaque (GE) or Lymphoprep (StemCell Technologies). European PBMCs were washed twice with PBS, then seeded in six-well culture plates (Corning) coated with 50 μg/mL rat tail collagen type 1 (BD Biosciences). Medium was changed every second day once colonies (from day 6 post-seeding) started to appear. Following isolation, PBMCs from the Ghanaian children were washed twice with PBS, and suspended in 350 μL EGM-2 plus Bullet Kit (Lonza) supplemented with 10% FBS, 50 μL DMSO, and 100 μl FBS. PBMC from the Ghanaian children were frozen using a Mr. Frosty^TM^ freezing container and stored overnight at -80˚C, then transferred to liquid nitrogen. Following transfer to Denmark, the frozen PBMC were thawed, washed in pre-warmed EGM-2 and processed as above. All experiments described in this paper were carried out with BOECs at passage 2 to 8.

### *P*. *falciparum in vitro* culture and selection

Erythrocytes infected by *P*. *falciparum* 3D7, FCR3/IT4, and HB3 were cultured *in vitro* using standard methodology [25]. The cultures were selected for IE surface expression of the PfEMP1 proteins PFD1235w, IT4VAR13, and HB3VAR03, respectively by panning with specific antibodies as described previously [25–27]. The genotypic identity of each clone was routinely verified by genotyping [28], and *Mycoplasma* infection regularly excluded using the MycoAlert *Mycoplasma* Detection Kit (Lonza) according to the manufacturer’s instructions.

### Immunofluorescence microscopy

To evaluate expression of endothelial adhesion receptors, BOECs isolated from European individuals at passage 3, were grown to confluence on 12 mm diameter glass coverslips pre-coated with 50 μg/mL collagen type 1 submerged in medium (EGM-2) in 24-well plates. Expression was evaluated on resting cells and on cells activated *in vitro* by TNF-α (Sinobiological) (10 ng/mL, 24 h). The BOECs were then fixed in ice-cold ethanol (10 min), blocked with PBS plus 2% FCS (30 min), followed by two washes (5 min each) with 500 μL PBS. The coverslips were then incubated with primary antibody (0.5 μg/well; 1 h), washed twice with PBS, and then incubated in the dark (45 min) with FITC-conjugated secondary antibody (1:500). Goat IgG anti-EPCR (R&D Systems), mouse IgG anti-ICAM-1 (clone 15.2, R&D Systems), mouse IgG anti-thrombomodulin (R&D Systems), mouse IgG anti-PECAM-1 (R&D Systems), mouse IgG anti-CD36 (clone FA6.152, Becton Dickson), mouse IgG anti-P1H12 (Biolegends) were used as primary antibodies. FITC-anti-mouse-IgG or FITC-anti-goat-IgG (EPCR, DAKO) were used as secondary antibodies. In addition, FITC-conjugated IgG anti-prominin-1 (CD133 (AC133); Milteyni Biotec) was used to label cells. After a final PBS wash (5 min), the coverslips were mounted cell-side down on a microscope slide with 5 μL of Prolong Gold anti-fade (Invitrogen). Images were captured with a Leica fluorescent microscope.

### Flow cytometry

*P*. *falciparum* IEs were DNA-labelled with ethidium bromide and surface-labelled with rat antibodies (1:40) specific for 3D7 PFD1235w, IT4VAR13, and HB3VAR03 as described (26), followed by FITC-conjugated secondary rabbit anti-rat IgG (1:150, Vector labs). Fluorescence FITC data from ethidium bromide-positive cells were collected on a Cytometic FC500 MPL flow cytometer (Beckman Coulter), and analysed using Winlist software (Verity House).

Resting or TNF-α-activated (10 ng/mL overnight) BOECs between passage 3–6 were detached with trypsin-EDTA, washed with DPBS, and suspended in FACS buffer (DPBS plus 3% BSA). The cells were incubated (45 min; 4˚C) with primary antibody, washed with assay PBS plus 3% BSA, incubated (30 min; 4˚C) with FITC-conjugated secondary antibody, and analysed by flow cytometry as above. Mouse monoclonal antibodies against human MUC18 (CD146; clone P1H12, Biolegend), PECAM-1 (CD31; clone 9G11, R&D systems), ICAM-1 (CD54; clone BBIG-11, R&D systems), thrombomodulin (CD141; clone 501733, R&D systems), platelet glycoprotein 4 (CD36; clone FA6.152, Beckman Coulter), and goat polyclonal antibody against human EPCR (CD201; polyclonal R&D systems) were used as primary antibodies. FITC-conjugated anti-mouse (Vector Laboratories) and anti-goat (Vector Laboratories) antibodies were used as secondary reagents. In addition, FITC-conjugated mouse anti-human CD133 was used.

### Adhesion assays under physiological flow conditions

BOECs between passage 3–6 were seeded onto micro-slides VI^0.4^ (Ibidi; 5x10^5^ cells per slide) pre-coated with rat tail collagen type 1 (50 μg/mL) and grown to confluence (≥ 48 h). Half of the channels were activated with TNF-α (10 ng/mL, 24 h) prior to assaying. IEs (3–5% parasitemia; 1% hematocrit; RPMI-1640 supplemented with 2% normal human serum) were flowed over the slides at microvascular wall shear stress (1 dyn/cm^2^, 5 min), and the number of bound IEs per mm^2^ assessed for a minimum of three independent experiments, performed in triplicate as described previously (2).

### Statistics

Data was analysed and statistical significance determined by performing paired t test in GraphPad Prism version 7.

## Results

### Establishment and characterization of BOEC cultures from fresh and frozen blood samples

**Fig 1:**
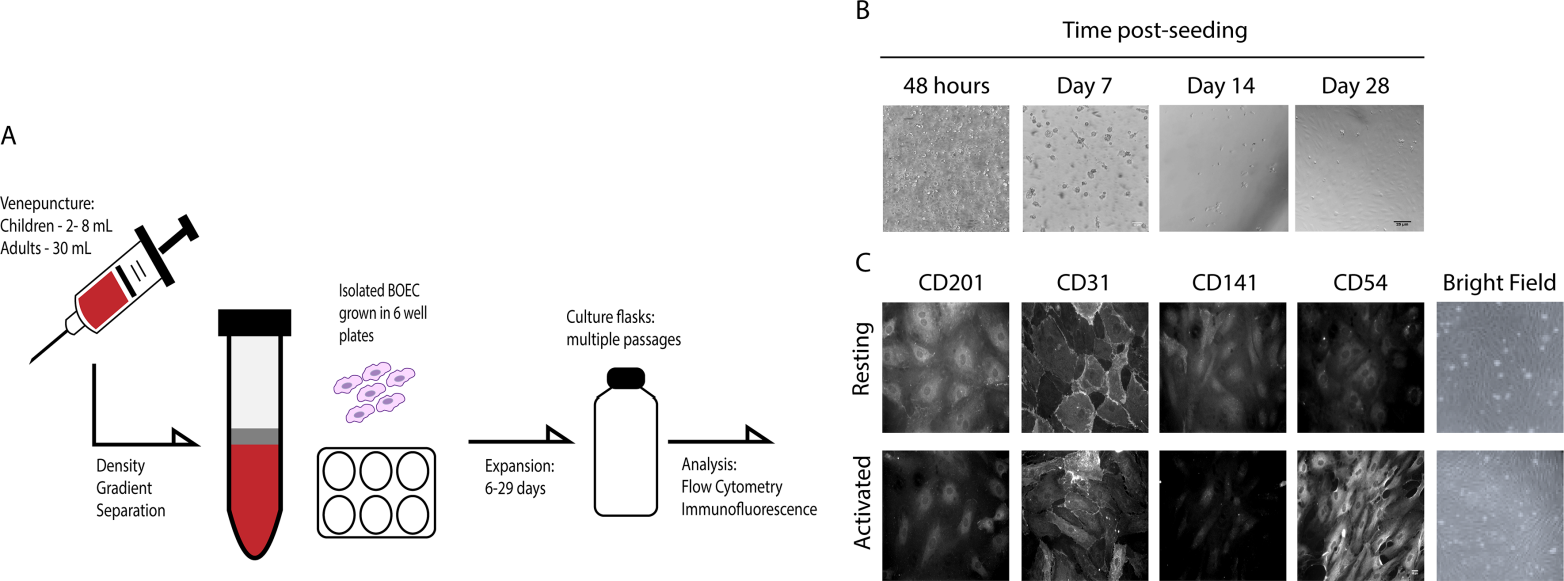
Establishing Blood Outgrowth Endothelial Cell (BOEC) lines from peripheral blood samples. (A) Process of isolating BOECs from human blood samples. B) Bright field images showing 48 hours post-seeding and documenting the appearance of progenitors (black arrow, day 7), small colonies (white arrow, day 14) and confluent BOEC (day 28) over time. C) Receptor expression (CD201/EPCR; PECAM-1/CD31; thrombomodulin/CD141 and CD54/ICAM-1) on resting and activated BOECs at passage 3. Shown is a representative immunofluorescence image (400 times magnification at passage 3) from a single donor. Bright field image (200 times magnification) shows elongation of cells after TNFα treatment.

**Fig 2:**
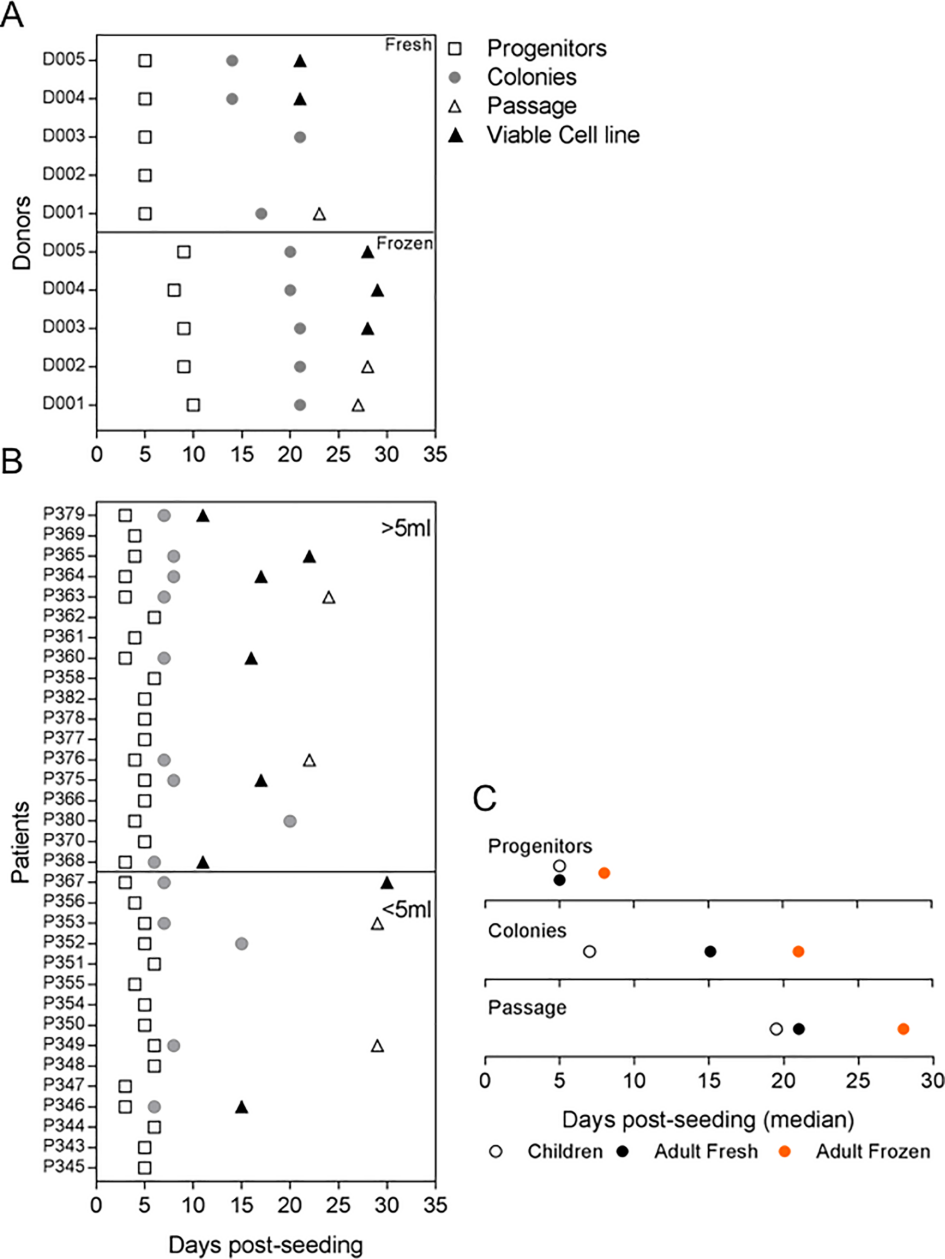
Blood Outgrowth Endothelial Cells (BOECs) from European adults and Ghanaian children. (A and B) Time (days) taken post-seeding for progenitor cells (□) and colonies (●) to appear and time to passage (day sub-culturing starts, △; ▲ denotes time to passage for donors who provided viable cells). BOECs were isolated from (A) European donors and (B) Ghanaian children. Patient data in B were grouped by blood volume: 2–5 mL (<5mL bottom panel) and 6–8 mL (>5ml top panel). (C) Appearance of progenitors and colonies and time to passage. Median days-post-seeding shown for each group; children (○), adult fresh samples (●) and adult frozen samples (●).

We used PBMC freshly isolated from large-volume (30 mL) samples of blood of healthy European adults to optimize the freeze and thaw technique for establishment of BOEC cultures, and for initial phenotypic characterization of the cells (Fig 1A). By day 7, spindle-like progenitor cells were observed in the PBMC cultures. These cells died off within 14 days, whilst colonies of BOECs with characteristic cobblestone-like morphology started to appear from day 13 in cultures from three of the five donors tested. The BOECs continued to grow, and gradually formed monolayers within the following week (Fig 1B). The BOECs were analysed via IFA for their surface receptor expression at passage 3, and found to be positive for the adhesion receptors EPCR, ICAM-1, thrombomodulin and PECAM-1 (Fig 1C). Activation of the BOECs in vitro by TNF-α resulted in increased expression of ICAM-1 (Fig 1C). Samples from the same donors were used to isolate BOECs from PBMC samples that had been frozen and stored in liquid nitrogen for one week. PBMCs were then thawed, seeded onto collagen-coated plates, and the appearance of progenitor colonies and time to passage recorded (Fig 2A). Like the fresh samples, all the frozen samples gave rise to progenitors, albeit slightly delayed (5–10 days versus 3–5 days). This delay in growth (Fig 2A and 2C) was also evident with respect to the appearance of colonies (median: 21 days) and for the time taken to reach passage (median: 28 days). We thus demonstrate isolation of BOECs from frozen samples and confirm previous reports describing establishment of BOECs from large-volume, fresh peripheral blood samples from adults [17,18,24].

### Establishment of BOEC cultures from small-volume, frozen PBMC samples

The main purpose of this study was to develop a protocol to establish BOEC cultures from children with malaria to provide a clinically relevant source of endothelial cells for the study of IE adhesion *in vitro*. This required adaptation of the above approach to small-volume (2–8 mL) blood samples and the use of cryopreserved rather than the freshly isolated PBMC used in previous studies [17,19,24,29]. We observed spindle-like endothelial progenitor cells within 72 hours of *in vitro* culture of cryopreserved PBMC from 33 of our Ghanaian malaria patients (of the original 34, one sample was discarded due to bacterial contamination). As expected, the progenitor cells died off within the next 14 days, while small colonies of BOEC cells started to appear from day 6 onwards (Fig 1B). Cultures of cells from 14 of the donors progressed to form confluent monolayers of ‘cobblestone-like’ endothelial cells. Of these, eight resulted in stable BOEC colonies that were able to expand. The overall success rate (8/33) of BOEC culture establishment from small-volume frozen PBMC samples was thus somewhat lower than from large-volume fresh samples (3/5). The success rate with the Ghanaian samples appeared to depend on initial sample size, as 6/9 cultures originated from blood samples >5 mL, compared to 2/5 from smaller-volume samples (Fig **2**A and **2**B, Table 1). We conclude that it is possible to establish BOEC cultures from small-volume samples of frozen PBMCs (Fig 2A–2C, Table 2).

**Table 1:** Summary of the growth and success rate of BOEC isolated from Ghanaian children.

Number of patients	34
Age in years	5.5 (3.75; 9)^a^
Gender (male)	17/34 (50%)
No of donors providing colonies	14/33 (42.4%)
success rate in ≤ 5 years	64.30%
Cells discarded^b^	1/34 (2.9%)
Days to appearance of cells:	
Within 8 days	9/14 (64.3%)
After 8 days	5/14 (35.7%)
Providing viable cell lines	8/14 (57.1%)

^a^median age with 25% and 75% percentiles

^b^one donor discarded due to contamination

**Table 2:** Median values for BOEC timeline.

	Median*		
	Children	Adult Fresh	Adult Frozen
Progenitors	5 (3.5–5)	5 (5–5)	9 (8.5–9.5)
Colonies	7 (7–8)	15.5 (14–20)	21 (20–21)
Passage	19.5 (15.25-27-75)	21 (21–23)	28 (27.5–28.5)

*Median with 25% and 75% percentiles shown in brackets.

### Expression of IE adhesion receptors by BOECs from paediatric malaria patients

**Fig 3:**
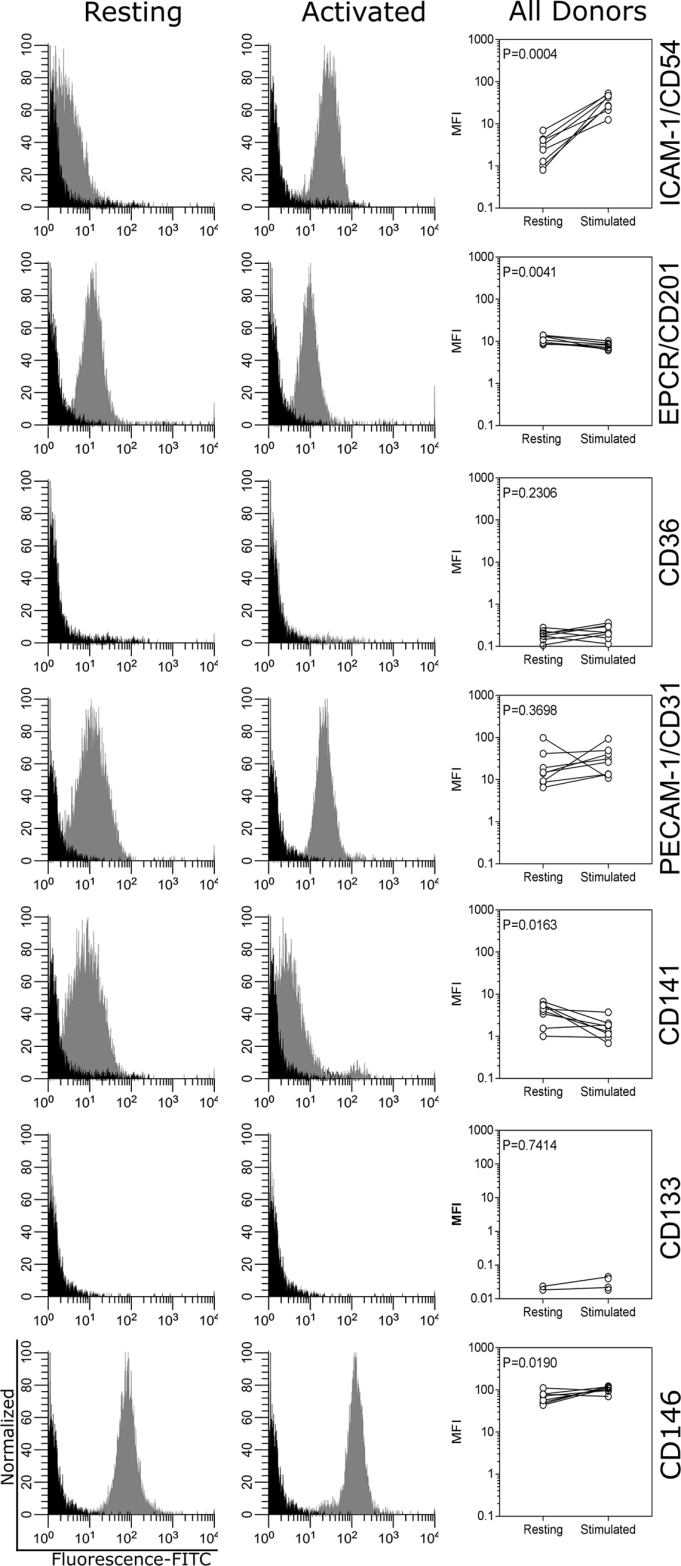
Flow cytometry (FACS) analysis of surface receptor expression. Surface expression of ICAM-1/CD54, EPCR/CD201, CD36, PECAM-1/CD31, CD141, MUC18/CD146 and prominin-1/CD133 on resting (grey histograms, left column) or TNFα-activated BOECs (grey histograms, middle column) and secondary only (black histograms, left and middle column) from one representative Ghanaian child (P360; passage 3–6). Right column: mean fluorescence intensity (MFI) of eight individual BOEC cells lines (passage 3–6) either resting or TNFα-activated. Data from three independent experiments performed in duplicate.

BOEC’s derived from cryopreserved small-volume PBMC samples from Ghanaian malaria patients (n = 8) all expressed EPCR, ICAM-1, thrombomodulin and PECAM-1, (Fig 3). The cells did not express CD133/prominin-1 (a stem cell marker), confirming that they were not progenitor cells; and were all positive for the endothelial cell marker CD146/MUC18 (Fig 3). The immunofluorescence assays demonstrated all observed cells expressing PECAM-1 around the periphery of each cell (representative image shown in Fig 1C). The positive signal for both PECAM-1 and CD146/MUC18 confirm the cells are endothelial in nature. Activation of the BOECs *in vitro* by TNF-α resulted in significant increases in the expression of ICAM-1 (P = 0.0004) and decreased expression of EPCR (P = 0.004) (Fig 3). FACS was similarly used to measure expression of additional known adhesion receptors (CD31/PECAM-1 and CD141/Thrombomodulin), and all BOEC cell samples tested positive. BOEC´s from none of the donors expressed CD36 (Fig 3) and all donor cell lines displayed altered morphology in response to TNF-α, with cells changing from characteristic cobble-stone morphology, to slightly elongated (Fig 1).

### BOECs support adhesion of *P*. *falciparum*-infected erythrocytes

**Fig 4:**
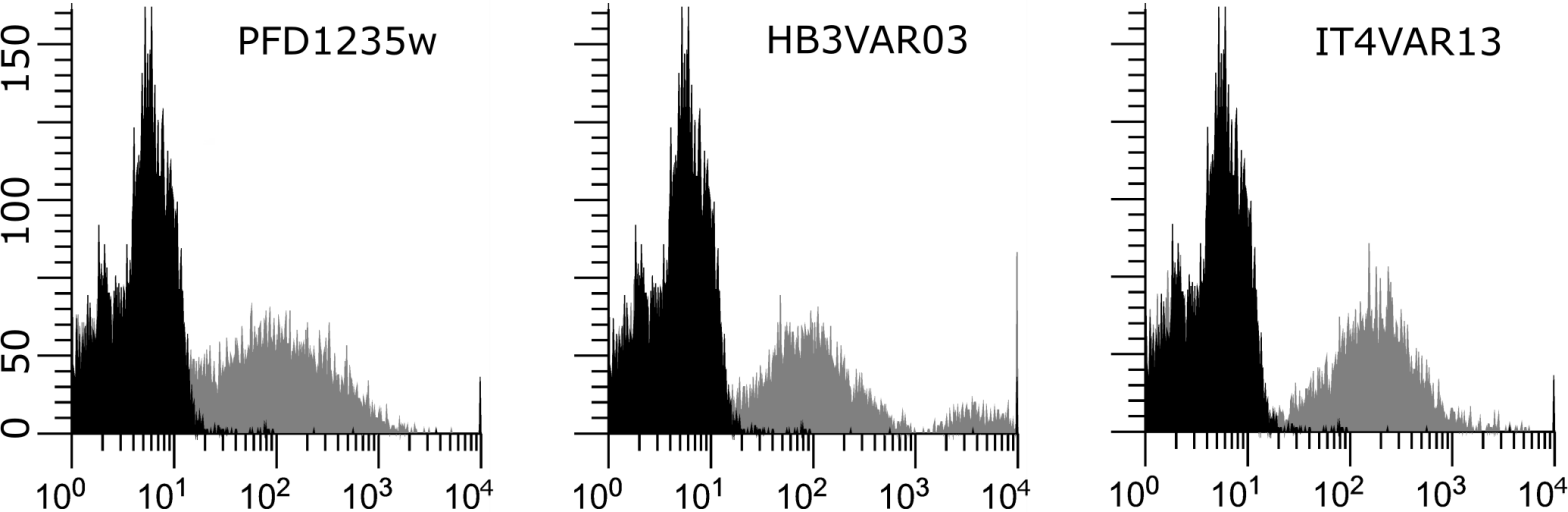
Flow cytometry analysis of *P*. *falciparum* infected erythrocytes for expression of specific PfEMP-1. The parasite lines 3D7 PFD1235w, HB3VAR03 and IT4VAR13 were stained with specific rat anti-sera (grey histograms) and non-specific rat sera (black histograms).

One BOEC cell line (Ghanaian donor P360, passage 3–6) was selected at random for analysis of the ability to support adhesion of *P*. *falciparum* IE´s under physiological flow conditions. Resting BOECs supported the adhesion of all the three parasite lines tested (HB3VAR03 IE, IT4VAR13 IE, PFD1235w IE). PFD1235w-positive IEs adhered equally well to both resting and TNF-activated BOECs, while IEs positive for HB3VAR03 or IT4VAR13 showed increased (statistically non-significant; P = 0.1158) levels of adhesion upon activation of the BOECs with TNF-α (Table 3, Fig 4.).

**Table 3:** Infected erythrocytes bound to BOECs.

Number IE bound per mm^2^						
	IT4VAR13		HB3VAR03		PFD1235w	
Patient ID	Resting	Activated	Resting	Activated	Resting	Activated
P360	20.8 ± 16.2^a^	107.4 ± 41.4	14.3 ±6.3	20.5 ±13.0	22.4 ±24.9	21.8 ±16.4

^a^ ± s.d. for three independent experiments, performed in triplicate of BOEC (passage 3–6).

## Discussion

To the best of our knowledge, this is the first report of successful isolation of viable BOEC cells from small blood volumes (≤ 8 ml) following cryopreservation. The BOECs were shown to express known *P*. *falciparum-*IE adhesins associated with cerebral disease (ICAM-1/CD54 and EPCR/CD201) [2,4], and levels of expression were altered in response to TNF-α, a cytokine associated with severe malaria[30]. The BOECs were able to support the adhesion of *P*. *falciparum* isolates, with increased IE adhesion following cytokine-induced activation. The isolates used (HB3VAR03, IT4VAR13, PFD1235w) are known ICAM-1 binders, and levels of adhesion were comparable to their previously reported adhesion to primary human brain microvascular endothelial cells [31].

Full understanding of the interactions between *P*. *falciparum* IE and the host endothelium is crucial for understanding the pathology of malaria infections. In the absence of suitable animal models for IE sequestration, investigators, including ourselves, have been using cells of non-human origin [32,33]; cells derived from non-African donors [16] from human tumours [14,15], or from commercially available sources as *in vitro* adhesion models. However, such cell lines may not accurately represent clinically relevant tissue, and we therefore propose BOEC cells as a clinically relevant alternative cell source. The BOEC model allows for the relatively straightforward acquisition of endothelial cells from different patient categories, and subsequent characterization of individual patient endothelium and investigation of *P*. *falciparum* IE adhesion. BOEC cells have been shown to maintain stable gene expression up to passage 15 for a variety of receptors [29]. A potential limitation is the lack of CD36 expression found amongst the donors tested. The BOEC analysed in this study did not express any CD36 (Fig 3), however CD36 expression is tissue specific and in many cases, CD36 is expressed by a sub-population of BOEC cells at different levels[17]. Whilst CD36 is indeed a common parasite adhesion receptor[34], it is not associated with severe malaria in Africa[35–37].

Access to clinically relevant tissue has until now relied upon biopsy or post-mortem samples, which are costly, time-consuming, and require technical expertise that is not always available. Wassmer *et al*. reported the successful isolation of endothelial cells from tissue biopsies from patients suffering from malaria[38]; this method is somewhat more invasive and involves an additional procedure to isolate cells. In addition to the practical aspects of sample collection, there is the issue of ethics and consent. In contrast, BOECs can be readily grown from a blood sample drawn using a minimally invasive method. Collection of small-volume peripheral blood samples in a field work setting is clearly feasible, and the BOEC method could therefore be added to currently approved protocols, providing easy access to a clinically relevant source of endothelial cells. Our recent publication identifying a subset of dual ICAM-1- and EPCR-binding *P*. *falciparum*-IEs associated with cerebral malaria indicates that important components in malaria pathogenesis may lie with the endothelium [2,39]. In these studies, some patients with severe disease seemingly carry dual binding IEs associated with cerebral disease, yet do not present with clinical symptoms of cerebral malaria. Based on our current knowledge we cannot know if a potential delay in seeking hospital treatment would have resulted in later admission with cerebral malaria, and/or if there is an as-yet unidentified host component that works in tandem with dual-adhesive IEs resulting in the potentially life-threatening manifestations of cerebral malaria. BOEC cells may provide insights not only into the state of the endothelium during severe disease, but also present the opportunity to investigate inter-individual and population-level differences. Differences that may explain why some children develop cerebral malaria, while other children do not.
